# Estimating nutrient concentrations and uptake in rice grain in sub-Saharan Africa using linear mixed-effects regression

**DOI:** 10.1016/j.fcr.2023.108987

**Published:** 2023-08-01

**Authors:** Tovohery Rakotoson, Kalimuthu Senthilkumar, Jean-Martial Johnson, Ali Ibrahim, Job Kihara, Andrew Sila, Kazuki Saito

**Affiliations:** a Laboratoire des RadioIsotopes (LRI), Université d′Antananarivo, BP 3383, Route d′Andraisoro, 101, Antananarivo, Madagascar; b Africa Rice Center (AfricaRice), P.O.Box 1690 Ampandrianomby, Antananarivo, Madagascar; c Africa Rice Center (AfricaRice), 01 B.P. 2551, Bouaké 01, Cote d′Ivoire; d University of Bonn, Institute of Crop Science and Resource Conservation (INRES), D-53115 Bonn, Germany; e Africa Rice Center (AfricaRice), Regional Station for the Sahel, B.P. 96, Saint-Louis, Senegal; f Alliance of Bioversity International and the International Center for Tropical Agriculture, c/o ICIPE Duduville Complex, Off Kasarani Road, P.O. Box 823-00621, Nairobi, Kenya; g World Agroforestry Centre (ICRAF), P.O. Box 30677, Nairobi 00100, Kenya

**Keywords:** Agro-ecological zone (AEZ), Production systems, Mineral fertilizer, Soil properties

## Abstract

**Context or problem:**

Quantification of nutrient concentrations in rice grain is essential for evaluating nutrient uptake, use efficiency, and balance to develop fertilizer recommendation guidelines. Accurate estimation of nutrient concentrations without relying on plant laboratory analysis is needed in sub-Saharan Africa (SSA), where farmers do not generally have access to laboratories.

**Objective or research question:**

The objectives are to 1) examine if the concentrations of macro- (N, P, K, Ca, Mg, S) and micronutrients (Fe, Mn, B, Cu) in rice grain can be estimated using agro-ecological zones (AEZ), production systems, soil properties, and mineral fertilizer application (N, P, and K) rates as predictor variables, and 2) to identify if nutrient uptakes estimated by best-fitted models with above variables provide improved prediction of actual nutrient uptakes (predicted nutrient concentrations x grain yield) compared to average-based uptakes (average nutrient concentrations in SSA x grain yield).

**Methods:**

Cross-sectional data from 998 farmers’ fields across 20 countries across 4 AEZs (arid/semi-arid, humid, sub-humid, and highlands) in SSA and 3 different production systems: irrigated lowland, rainfed lowland, and rainfed upland were used to test hypotheses of nutrient concentration being estimable with a set of predictor variables among above-cited factors using linear mixed-effects regression models.

**Results:**

All 10 nutrients were reasonably predicted [Nakagawa’s *R*^*2*^ ranging from 0.27 (Ca) to 0.79 (B), and modeling efficiency ranging from 0.178 (Ca) to 0.584 (B)]. However, only the estimation of K and B concentrations was satisfactory with a modeling efficiency superior to 0.5. The country variable contributed more to the variation of concentrations of these nutrients than AEZ and production systems in our best predictive models. There were greater positive relationships (up to 0.18 of difference in correlation coefficient *R*) between actual nutrient uptakes and model estimation-based uptakes than those between actual nutrient uptakes and average-based uptakes. Nevertheless, only the estimation of B uptake had significant improvement among all nutrients investigated.

**Conclusions:**

Our findings suggest that with the exception of B associated with high model EF and an improved uptake over the average-based uptake, estimates of the macronutrient and micronutrient uptakes in rice grain can be obtained simply by using average concentrations of each nutrient at the regional scale for SSA.

**Implications:**

Further investigation of other factors such as the timing of fertilizer applications, rice variety, occurrence of drought periods, and atmospheric CO_2_ concentration is warranted for improved prediction accuracy of nutrient concentrations.

## Highlights

• Concentrations of 10 nutrients in rice grain in sub-Sahara Africa were estimated using LMER model.
• Predictors used were agro-ecological zone, production system, fertilizer rate, and soil properties.
• 10 nutrients were reasonably estimated with Nakagawa’s *R*^*2*^ ranging from 0.27 (Ca) to 0.79 (B).
• The estimation of K and B concentrations was satisfactory with a modeling efficiency > 0.5.
• Use of the above models did not improve the estimation of nutrient uptake except for B.

## 1 Introduction

Quantification of nutrient concentrations in rice grain allows for the evaluation of crop nutrient removal, nutrient use efficiency, soil nutrient balance, and future nutrient needs, and develop fertilizer recommendation guidelines (Fairhurst et al., 2007). Mineral nutrients in rice grain are essential for human nutrition (Du et al., 2013, Prom-u-thai et al., 2020) and easy access to such data can help to sufficiently produce rice with nutritionally superior quality while reducing environmental footprints (Johnson et al., 2021). While it is relatively easy to collect data on rice yield in the fields, the determination of crop nutrient concentrations from which nutrient uptakes and removal are obtained requires costly and slow laboratory analyses or rapid and accurate spectroscopy techniques. As for the latter, it is well established that near-infrared (NIR, 700–2500 nm) and mid-infrared (MIR, 2500–16,670 nm) can accurately predict nutrient concentrations in plants (Reeves, 1994, Reeves and Van Kessel, 2002, Richardson and Reeves, 2005) . Despite the fact that spectroscopy techniques allow for faster and more cost-effective estimation of such data than conventional wet chemistry, in the long-term perspective, rapid, low-cost, and reliable estimation of nutrient concentrations and uptake in rice grains is urgently needed for the development of nutrient management frameworks in SSA.

Nutrient concentration and uptake in crops can vary from individual fields to whole countries and the world (Ludemann et al., 2022). Studies on cereal crops in various regions of the world suggest environmental factors (Du et al., 2013, Huang et al., 2016) such as climatic conditions (Dobermann and Fairhurst, 2000, Shimoyanagi et al., 2021), soil fertility level (Kekulandara et al., 2019), nutrient supply (Dobermann and Fairhurst, 2000) and crop management practices (Dobermann and Fairhurst, 2000, Tenorio et al., 2019) including fertilizer applications (Haefele et al., 2022, Ludemann et al., 2022, Tenorio et al., 2019) and crop genotype (Huang et al., 2016, Pinson et al., 2015) are the most reported factors impacting variations of nutrient concentrations in grain. For the case of SSA, recent results obtained from on-farm surveys observed agroecological zones (AEZ - (HarvestChoice, 2015)) and production systems which can be both categorized as environmental factors, as the main factors affecting most variations in the concentration of macronutrients and micronutrients in rice grain (Johnson et al., 2021).

Remobilization of nutrients plays a major role in their accumulation in crop grains and is particularly important during grain formation/filling (White, 2012). The extent of this nutrient remobilization during the reproductive stage depends on various factors, including the specific nutrient requirements, nutrient status of vegetative parts, and nutrient uptake rate by roots and this latter is largely dependent on the availability of nutrients in the soil. The bioavailability of all nutrients in the soil and the rhizosphere depends on some key soil physical and chemical properties and together with the forms/speciations of each nutrient, both can influence their transport and susceptibility for root uptake and consequently their accumulation in plant tissue including the grains (Russell et al., 2006, Stangoulis and Knez, 2022, Zone et al., 2020). Mineral N, P, and K fertilizers use can raise the concentrations of macronutrients such as P, K, Mg, and S (Haefele et al., 2022) and so for Ca concentration with only mineral N application (Lin et al., 2014). Concentrations of micronutrients such as B, Mn, and Cu in rice grain were also shown to be increased by mineral fertilizer use (Hao et al., 2007, Kuppusamy et al., 2017). In addition, mineral N, P and K fertilizers use could enhance Fe accumulation in rice grain (Kuppusamy et al., 2017, Stangoulis and Knez, 2022), but excessive applications of these nutrients have been reported to cause the opposite effect (Panda et al., 2012, Su et al., 2018). In SSA, how significant specifically soil physical and chemical properties and mineral fertilizer application as crop management factors would contribute to the variation of nutrient concentrations in rice grain remains to be further evaluated.

In SSA, Johnson et al. (2021) reported that among around two thousand surveyed fields, the Fe concentration of rice grain was particularly low compared to other nutrients. Micronutrient deficiency or hidden hunger in humans has been an important study area/topic in various health problems in regions where rice is a staple food (Prom-u-thai et al., 2020). Although the deficiency in micronutrients, mostly Fe, Zn, iodine (I), and selenium (Se) affects population health in different ways (Bailey et al., 2015, Harding et al., 2018), Fe deficiency has been reported as the most common among them (Bailey et al., 2015). Iron deficiency is often associated with anemia which affects more than one billion people worldwide (Bailey et al., 2015, Camaschella, 2019). The prevalence of Fe deficiency anemia in SSA is high and differs from the rest of the world partly because of low Fe dietary habits (Mwangi et al., 2021). At the international level, efforts have been engaged for the development of micronutrient-enriched or biofortified crops as a strategy for hidden hunger alleviation, and rapid and accurate analysis of micronutrient concentrations is essential for this purpose (Stangoulis and Knez, 2022). Zinc is an important micronutrient and is included in rice biofortification targets but the determination of its concentration in plant tissue spectroscopically – a method with which our data was partly obtained – was not as good as for the other above-cited nutrients (Johnson et al., 2021).

**Fig. 1:**
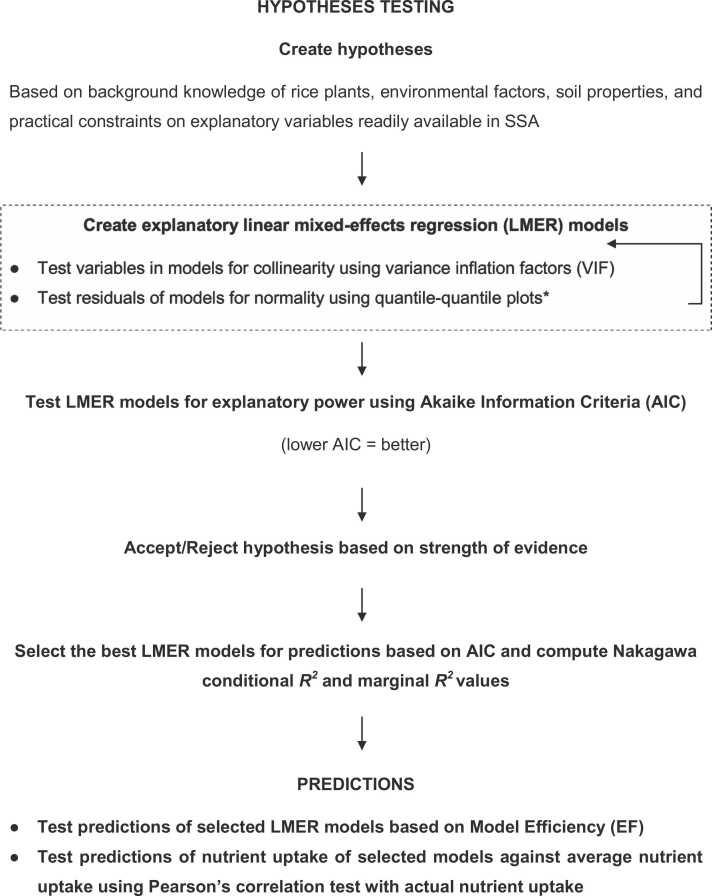
Workflow for investigating effects of agroecological zones, production system, soil properties, and crop management on the variation of nutrient concentrations in rice grain in SSA. *Residuals from Fe prediction models were not normally distributed but will not influence the outcome with the large samples > 10 observations per variable (Schmidt and Finan, 2018).

This study aimed to assess the influence of environmental (AEZ, production systems, and soil properties) and crop management (mineral fertilizer use) factors on the variations of macro- (N, P, K, Ca, Mg, S) and micronutrient (Fe, Mn, B, Cu) concentrations in rice grain in SSA and to assess whether these concentrations can be estimated using a limited set of variables within categories of these two factors. It was attempted to identify if estimation-based nutrient uptakes have greater relationships with actual nutrient uptakes than average-based uptakes. A modified workflow used by Ludemann et al. (2022) was implemented toward our aims in which the hypotheses were elaborated based on key bases allowing us to define which of the above factors are expected to influence nutrient concentrations of rice grain (Fig. 1).

(adapted from Ludemann et al., 2022).

## 2 Materials and methods

### 2.1 Study site and data collection

Data were collected during farmer’s field surveys between the 2012 – 2015 wet seasons, through an agronomy research network called “the Africa-wide Rice Agronomy Task Force”, led by the Africa Rice Center (AfricaRice) and involving more than 20 national agricultural research institutes (NARIs) in SSA. The selection of study sites, sampling methods, key agronomic practices, and environmental conditions (weather, soil) are described by Tanaka et al. (2017), Dossou-Yovo et al. (2020), and Senthilkumar et al. (2020). The sampling and processing of plant samples are described in Johnson et al. (2021), and those of soil samples in Johnson et al. (2019).

**Fig. 2:**
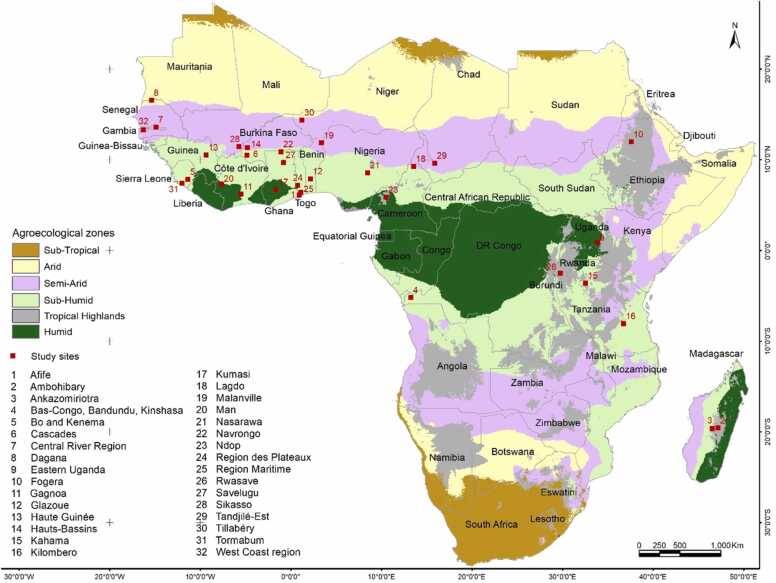
The 32 survey sites in 20 sub-Saharan African countries overlaid on agro-ecological zones (HarvestChoice, 2015).

In these surveys, 1628 farmers’ fields were originally investigated at 34 sites and in 20 countries (Johnson et al., 2021). After data cleaning and removal of missing values, the total number of fields used for this study was 998 from 32 sites (Fig. 2) across the original 5 AEZs (arid, semi-arid, humid, sub-humid, and highlands) in SSA (HarvestChoice, 2015); and three production systems (irrigated lowland, rainfed lowland, and rainfed upland). The number of surveyed fields per AEZ was: 36 (arid) and 264 (semi-arid) which were merged to 300 and referred to as arid to have more equilibrated numbers, 106 (humid), 423 (sub-humid), and 169 (highlands); and per production systems: 326 (irrigated lowland), 438 (rainfed lowland) and 234 (rainfed upland).

Nutrient concentrations (N, P, K, Ca, Mg, S and Fe, Mn, B, Cu) in rice grains were estimated with NIR-MIR infrared spectroscopy, as described in Johnson et al. (2021). In brief, NIR-MIR diffuse reflectance spectroscopy data were obtained using a Fourier-transform infrared spectrometer. Then, second-derivative spectra were fitted against plant laboratory reference data using partial least-squares regression (PLSR) to estimate the macronutrient and micronutrient concentrations in the rice grain samples (Johnson et al., 2021). Standard laboratory methods for the development of calibration and validation models were Kjeldahl acid digestion (Jones, Jr, 2001) for N, P, K, Ca, and Mg; and microwave digestion with nitric acid and hydrochloric acid and assayed in inductively coupled plasma – optical emission spectrometry (ICP-OES) (Miller et al., 2013) for S, Fe, Mn, B, Cu. As mentioned in the introduction, Zn concentration was not included in this study because of its poor estimation with the spectroscopy techniques.

Details on soil analysis methods are available in Johnson et al. (2019). Soil TOC, total N, exchangeable elements (K, Ca, Mg, Mn, Cu, B, and Al), sum of exchangeable cations (Exch-Cations), and CEC were estimated with combined NIR-MIR diffuse reflectance spectroscopy while the other parameters were determined with conventional wet chemistry. Standard laboratory methods for the development of calibration and validation models are as follow: soil TOC and total N – dry combustion (McGeehan and Naylor, 1988), exchangeable elements (K, Ca, Mg, Mn, Cu, B, and Al) with Mehlich-3 extractant (Gavlak et al., 2005), sum of exchangeable cations (Exch-Cations) with Mehlich-3 extractant (Gavlak et al., 2005), and CEC extracted with Mehlich-3 (Ross, 1995). Soil pH was determined in water (H_2_O) with soil:water ratio of 1:2.5 (Mclean, 1982). Exchangeable P was extracted with the Bray-2 method (Bray and Kurtz, 1945) and the clay content (Clay) was determined with the particle size distribution by sedimentation – pipette method (Gee and Bauder, 1986).

Using a standardized field survey protocol consisting, among others, of structured interviews with farmers and field monitoring, mineral N, P, and K fertilizers application rates were obtained (Tanaka et al., 2017).

### 2.2 Hypothesis and prediction accuracy testing

Our hypothesis was that concentrations of macro- (N, P, K, Ca, Mg, S) and micronutrients (Fe, Mn, B, Cu) in rice grain in SSA can be parsimoniously (economically) estimated with AEZ, production systems, soil properties and mineral fertilizer application rates (Fig. 1).

For the hypothesis testing, linear mixed-effects regression (LMER) models were used in which “Country” was entered as a random factor to account for inter-country variability while examining the specific effects of the above variables of interest listed in Table 1. Next, the Modeling Efficiency (EF - Nash and Sutcliffe (1970)) criterion was used to assess the prediction accuracy of the selected models. Additionally, the relationships between predicted nutrient uptake from best-fitted models and the average nutrient uptake with actual nutrient uptake were compared through Pearson’s correlation test.

**Table 1:** Relevant variables for hypothesis testing to predict rice nutrient concentrations in this study dataset.

Category	Abbreviation	Description	Units
**Predictor variables**			
Environment	AEZ	Agro-ecological zones	
	Production systems	Production systems	
Soil	pH	pH water	
	N	Total nitrogen (N)	%
	TOC	Total organic carbon (C)	%
	Bray-P	Available phosphorus (P), Bray-2 method	mg kg^−1^
	Clay	Clay content	%
	K-exch	Exchangeable potassium (K) - K^+^, Mehlich-3 method	g kg^−1^
	Ca-exch	Exchangeable calcium (Ca) - Ca^2+^, Mehlich-3 method	g kg^−1^
	Mg-exch	Exchangeable magnesium (Mg) - Mg^2+^, Mehlich-3 method	g kg^−1^
	Mn-exch	Exchangeable manganese (Mn) - Mn^2+^, Mehlich-3 method	mg kg^−1^
	Exch-Cations	Sum of exchangeable cations	cmol(+) kg^−1^
	Cu-exch	Copper (Cu) – Cu^2+^, Mehlich-3 method	mg kg^−1^
	B-exch	Boron (B) - H_3_BO_3_ and H_2_BO_3_^-^, Mehlich-3 method	mg kg^−1^
	Al-exch	Aluminum (Al) – Al_3_^+^, Mehlich-3 method	g kg^−1^
	CEC	Cation Exchange Capacity, Mehlich-3 method	cmol(+) kg^−1^
Management	N rate	Inorganic N fertilizer application rate in the main field	kg N ha^−1^
	P rate	Inorganic P fertilizer application rate in the main field	kg P ha^−1^
	K rate	Inorganic K fertilizer application rate in the main field	kg K ha^−1^
**Dependent variables**			
Grain	N	N concentration	%
	P	P concentration	%
	K	K concentration	%
	Ca	Ca concentration	%
	Mg	Mg concentration	%
	S	Sulfur (S) concentration	%
	Fe	Iron (Fe) concentration	mg kg^−1^
	B	B concentration	mg kg^−1^
	Mn	Mn concentration	mg kg^−1^
	Cu	Cu concentration	mg kg^−1^

Each nutrient constituted one hypothesis for testing the explanatory power of the different models in Table 2. Predictor variables for model components were selected according to background knowledge (Heinze et al., 2018) which is described in the introduction section. Variables were also selected so that no multicollinearity issue occurs in the models. For all the 10 macro- and micronutrients investigated in this study, AEZ and production systems were used as starting base components of the models and as fixed factors.

**Table 2:** Model compositions for hypothesis testing of variables explaining N, P, K, Ca, Mg, S, Fe, B, Mn, and Cu concentrations in rice grain.^a^

Nutrients	Model ID	Model composition
***All***	Mod A1	AEZ
	Mod A2	AEZ+Production systems
	Mod A3	AEZ+Production systems+pH+N + TOC+Bray-P + Clay+K-exch+Ca-exch+Mg-exch+Mn-exch+Cu-exch+B-exch+Al-exch+CEC
	Mod A4	AEZ+Production systems+TOC
	Mod A5	AEZ+Production systems+Clay
	Mod A6	AEZ+Production systems+Exch-Cations
	Mod A7	AEZ+Production systems+CEC
	Mod A8	AEZ+Production systems+N rate+P rate+K rate
***Specific***		
**N**	Mod N9	AEZ+Production systems+N
	Mod N10	AEZ+Production systems+N rate
	Mod N11	AEZ+Production systems+N + N rate
**P**	Mod P9	AEZ+Production systems+Bray-P
	Mod P10	AEZ+Production systems+Al-exch
	Mod P11	AEZ+Production systems+Bray-P + Al-exch+P rate
**K**	Mod K9	AEZ+Production systems+K-exch
	Mod K10	AEZ+Production systems+K rate
	Mod K11	AEZ+Production systems+K-exch+K rate
**Ca**	Mod Ca9	AEZ+Production systems+Ca-exch
**Mg**	Mod Mg9	AEZ+Production systems+Mg-exch
**B**	Mod B9	AEZ+Production systems+B-exch
**Mn**	Mod Mn9	AEZ+Production systems+Mn-exch
**Cu**	Mod Cu9	AEZ+Production systems+Cu-exch

a Abbreviations for variables are described in Table 1. “Country” was used in all the models as a random effect of a mixed-effects model. AEZ: 5 reduced to 4 (arid and semi-arid combined) classes for sub-Saharan Africa (HarvestChoice, 2015). Production systems**:** irrigated lowland, rainfed lowland, and rainfed upland. "A” in Mod A1 to A8 means all nutrients.

Because of interactions between elements during uptake by plants (Liu et al., 2017), we included one model with predictor variables all soil properties available in our dataset but which did not have multicollinearity issues for all the evaluated soil properties and nutrients (pH, N, TOC, Bray-P, Clay, K-exch, Ca-exch, Mg-exch, Mn-exch, Cu, B, Al, and CEC). Then, followed by separate models constituted of TOC, Clay, Exch-Cations, and CEC. N, P, and K rates used altogether in one model. Additional models with soil N, N rate and their combination were tested separately for grain N. A similar approach was used for grain P and K with Bray-P and Al as soil attributes for the first one while only K-exch for the second, and P and K rates as crop management respectively. For grain Ca, Mg, B, Mn, and Cu, soil Ca-exch, Mg-exch, B, Mn-exch, and Cu were tested respectively as additional models. No additional model was tested for grain S and Fe.

#### 2.2.1 Statistical procedures for hypothesis testing

The multicollinearity test was performed by calculating the Generalized Variance-Inflation Factor (GVIF) (Fox and Monette, 1992) with the R software (“car” package) for all the models. The GVIF is more appropriate than the VIF if the model includes categorical variables leading to a degree of freedom > 1. As in Ludemann et al. (2022), GVIF< 10 was considered acceptable. The assumptions of normality and homoscedasticity of residuals were assessed with the normal quantile-quantile plots. Only grain Fe models did not meet the normal distributions of residuals but no data transformations were done to remedy this since our very large observations (>10 per variable) would nullify any influence of such on the outcomes (Schmidt and Finan, 2018). Interaction effects of explanatory variables were omitted to reduce the probability of introducing false inferences or type 1 errors (Ludemann et al., 2022).

Models were run with R using the “lmer” function (Bates et al., 2015). The Akaike Information Criterion (AIC) (Akaike, 1998) was computed using the Restricted Maximum Likelihood framework (REML) to compare the models, and the best (low AIC) but “parsimonious’ (most economical) approximating models were selected. The total variance explained was subsequently checked by calculating Nakagawa’s conditional (*R*^*2*^ cond.) coefficient of determination (Nakagawa et al., 2017) using the ”performance” package. This approach was used by Ludemann et al. (2022) and Munyai and Foord (2015) as the lowest AIC did not always correspond to the highest *R*^*2*^. Nakagawa’s conditional *R*^*2*^ estimates the variations accounted for in both fixed and random factors of a model; given that “Country” is the only random factor in all models.

#### 2.2.2 Methods for assessing prediction accuracy of selected models

For all models, prediction accuracy was evaluated with the modeling efficiency (EF) (Nash and Sutcliffe, 1970):

$$\mathrm{EF}=1-\sum \frac{{\left(S_{i}-O_{i}\right)}^{2}}{{\left(\overset{®}{O}-O_{i}\right)}^{2}}$$

Where *S*_i_ is the predicted concentration for nutrient *i*, *O*_i_ is the concentration for nutrient *i*, and *Ō* is the average of observed concentration. A value of 1 for EF indicates perfect prediction while a value of 0 means the model predicts no better than the mean of observations. Negative values suggest that the average of observed values is a better predictor than the model. Values of EF superior to 0.5 are considered satisfactory according to Moriasi et al. (2007).

### 2.3 Evaluation of the importance of predictor variables in best models

The importance of predictor variables was assessed with the amount of variance explained by a particular or a set of explanatory fixed-factor variables measured with the marginal *R*^*2*^ which was also computed using “performance” package (Lüdecke et al., 2021).

To further analyze the effect of fixed factors level, the coefficients of the estimates from the best models were generated and *p*-values computed with Kenward-Roger’s approximation of the degrees of freedom method for linear mixed models (Kenward and Roger, 1997) using the “lmerTest” package.

### 2.4 Evaluation of relationships between estimation-based nutrient uptakes with actual nutrient uptakes against average-based uptakes with actual nutrient uptakes

For each of the predictor variable combinations, relationships between (i) estimated nutrient uptakes (*predicted nutrient concentration x grain yield*) obtained with the best models and actual nutrient uptakes (*actual nutrient concentration x grain yield*) were compared with that of (ii) average nutrient uptakes (*average nutrient concentration for the whole data x grain yield*) and actual nutrient uptakes. Grain yield was 14%-adjusted for moisture content. For this, relationships were visually assessed together with Pearson’s correlation test (*p* < 0.05) where the coefficient of correlation (*R)’s* of (i) and (ii) were compared.

## 3 Results

### 3.1 Observed variations of dependent and predictor variables

#### 3.1.1 Nutrient concentrations in rice grain samples

Average values of concentrations in rice grain of all 10 nutrients for each country, and across AEZ and production systems are shown in Table 3, and coefficients of variation are shown in Table B1. Concentrations of Fe (CV = 100%), B (CV = 31%), Mn (CV = 29%), and Cu (CV = 48%) in rice grain have large variations. For the other nutrients, variations were rather small with CV below 23%.

**Table 3:** Mean values of N, P, K, Ca, Mg, S (%) and Fe, B, Mn, Cu (mg kg^−1^) concentrations in rice grain “n” farmers’ fields for each country, and across agro-ecological zones (AEZ) and production systems, and overall. Coefficients of variation (%) are between brackets for the overall data and are in supplementary information when not shown. Nutrient concentration values are displayed as colors ranging from green (lowest) to red (highest).

^**a**^ AEZ: 5 reduced to 4 classes (arid and semi-arid combined) for sub-Saharan Africa (HarvestChoice and (IFPRI), 2015). ^**b**^ n denotes the number of farmer’s fields. ^**$**^ Calculated based on Dobermann and Fairhurst (2000)

Concentrations varied rather largely between countries for all the 10 nutrients with the exception of Ca (0.03 – 0.05%). Concentrations of N in grain were below the average of 1.45% (CV = 14%) in Benin, Burkina Faso, Cameroon, Chad, DRC, Ethiopia, Guinea, Mali, Nigeria, and Rwanda. For P, concentrations were on average 0.25% (CV = 21%) and values below this average value were found in Benin, Burkina Faso, Chad, Côte d′Ivoire, Guinea (with the lowest value of 0.19%, CV = 17%), Mali, Niger, Nigeria and Sierra Leone. Concentrations of K were all very low and ranged from 0.24% (CV = 19%) in Togo to 0.45% (CV = 15%) in Madagascar. Concentrations of Ca ranged between 0.03% and 0.05% (CV = 11 – 22%) with the lowest value observed only in DRC. For Mg, concentrations were also low for all countries, and where lowest values of 0.09% (CV = 14 – 30) were for Guinea, Mali and Togo. Benin, Chad, DRC, Guinea, Mali, Rwanda, Tanzania, and Togo had S concentrations in rice grain lower between 0.08% and 0.09% (CV = 6 – 14%). Concentrations of Fe were lower in Burkina Faso, Côte d′Ivoire, DRC, Ghana, Niger, Nigeria, Senegal, Sierra Leone, Togo, and Uganda between 66 and 199 mg kg^−1^ (CV = 25 – 56%). For B, all countries’ values were low and ranged from 1.18 mg kg^−1^ for Guinea to 2.78 mg kg^−1^ for Senegal. Only DRC (35.31 mg kg^−1^, CV = 18%) and Guinea (45.28 mg kg^−1^, CV = 18%) had low Mn concentrations in rice grain among the 20 countries. Concentrations of Cu were very high ranging from 14.36 mg kg^−1^ (Côte d′Ivoire) to 27.30 mg kg^−1^ (Guinea) (CV = 11 – 68%).

Values for all the 10 nutrients presented no consistency and were spread across AEZ. Highlands was the only AEZ having low N concentrations in rice grain (1.34%, CV = 13%) while P concentrations were low for arid and sub-humid. Concentrations of K were obviously very low (0.31% in sub-humid to 0.38% in highlands). A similar trend is also observed for concentrations of Ca which were all low for all AEZ with the lowest value for humid (0.041%, CV = 14%); for Mg concentrations (<0.15%) and for B concentrations (5 mg kg^−1^) with this later lowest for humid (1.69 mg kg^−1^, CV = 31%). For S concentrations, there was a very small variation between AEZ with values of 0.10% with the exception of humid (0.11%). Concentrations of Fe were low for humid (124 mg kg^−1^, CV = 64%) and sub-humid (155 mg kg^−1^, CV = 83%). Concentrations of Mn and Cu in rice grain were both high overall but lower values were observed in arid and sub-humid (∼63 mg kg^−1^) for the first and in humid (16.2 mg kg^−1^, CV = 42%) for the second.

There was also no consistency between production systems for all the nutrients. Concentrations of N were low under rainfed lowland system (1.43%, CV = 14%), while P concentrations were low for both rainfed lowland and rainfed upland at 0.25% (CV = 23%) and 0.23% (CV = 18%), respectively. Again, K, Ca, Mg, and B concentrations were low for all three production systems. Concentrations of S in rice grain were not affected by production systems and were all equal to 0.10% (CV = 14 – 15%). For Fe, only rainfed upland had a value below 200 mg kg^−1^ (149 mg kg^−1^, CV = 88%). As observed above and across production systems, Mn and Cu concentrations were also very high for all three production systems but lowest for rainfed upland (60 mg kg^−1^, CV = 31%) for Mn while for the two rainfed production systems (∼19 mg kg^−1^) for Cu.

#### 3.1.2 Soil properties

Table 4 shows the average values of all selected soil properties in this study and coefficients of variation are shown in Table B2. In overall, all of them presented very large variations (CV between 55% and 130%) with the exception of pH (CV = 13%).

**Table 4:** Mean values of the relevant soil properties for this study of “n” farmers’ fields for each country, agro-ecological zones (AEZ) and production systems, and overall. Coefficients of variation (%) are between brackets for the overall data and are in supplementary information when not shown. Soil properties values are displayed as colors ranging from green (lowest) to red (highest).

^a^n denotes the number of farmer’s fields

For all countries, soils are acidic and pH values below 5 are seen in Burkina Faso, DRC, Niger, Nigeria, and Sierra Leone. Soil CEC was on average 11.76 cmol(+) kg^−1^ (CV = 85%) and Mali, Nigeria, and Sierra Leone had very low values below 6 cmol(+) kg^−1^. Overall, soils also had low TOC content which was on average 1.77% (CV = 87%); TOC values in Benin, Niger, Senegal, and Tanzania were lowest and below 1%. Clay content was on average 28.65% (CV = 65%) in overall with the lowest values below 20% observed in Benin, Chad, Nigeria, Tanzania, and Togo (10.89–19.64%). Soil N was on average 0.19% (CV = 90%) and countries including Benin, Mali, Niger, Nigeria, Senegal, Tanzania, and Togo had values below 0.1% (0.05–0.09%). On average, Bray-P was very low and varied a lot (3.65 mg kg^−1^, CV = 130%) and very low values below 2 mg kg^−1^ were in Chad, Niger, Rwanda, and Tanzania (0.27 – 1.84 mg kg^−1^). Such a low level of available P was already reported by Johnson et al. (2021), which used the same data set as this paper. Phosphorus deficiency can be attributable to P-fixation by kaolinite and adsorption on iron oxides (Gilkes and Prakongkep, 2016) and low application of mineral fertilizer and organic input (Haefele et al., 2022). Soil K-exch was on average 0.09 g kg^−1^ (CV = 55%) and was below 0.1 g kg^−1^ for most of the countries with the exception of Cameroon, Côte d′Ivoire, Ethiopia, Ghana, Madagascar, Senegal, and Uganda. With regards to Ca-exch (<0.4 g kg^−1^), Mg-exch (<0.1 g kg^−1^), Mn-exch (below or equal to 0.04 g kg^−1^), and Exch-Cations (<3.5 cmol(+) kg^−1^) lowest values were observed in Mali, Nigeria, and Sierra Leone. Cu-exch was on average 2.16 mg kg^−1^ (CV = 60%) and the lowest value for this element in soil was 1.12 mg kg^−1^ (CV = 28%) in Nigeria. B-exch varied a lot (CV = 108%) and was on average 0.08 mg kg^−1^, and countries including Burkina Faso, Chad, Mali, Niger, Nigeria, and Sierra Leone were associated with the lowest values (<0.05 mg kg^−1^). For Al-exch, the overall average was 7.93 mg kg^−1^ (CV = 58%) with the lowest value of 4.47 mg kg^−1^ (CV = 29%) for Nigeria and the highest of 15.85 mg kg^−1^ (CV = 42%) for Sierra Leone.

No single AEZ was found to be associated with the highest or lowest values for all soil proprieties across the different production systems. Variation of soil pH was minimal and the lowest value belonged to highlands (5.31, CV = 12%). Soil CEC was lower (<12 cmol(+) kg^−1^) in the other three production systems compared to that of highlands. Values of TOC (1.07%, CV = 58%), N (0.09%, CV = 56%), K-exch (0.08 g kg^−1^, CV = 50%), Ca-exch (0.94 g kg^−1^, CV = 67%), and Mn-exch (0.06 g kg^−1^, CV = 67%) were all lowest for arid with the later (i.e Mn-exch) having low equal value for sub-humid. Clay (22.12%, CV = 40%), Mg-exch (0.19 g kg^−1^, CV = 105%), Exch-Cations (7.08 cmol(+) kg^−1^, CV = 83%), Cu-exch (1.99 g kg^−1^, CV = 40%), and Al-exch (6.13 g kg^−1^, CV = 43%) were lowest for humid. Bray-P was lowest for highlands (2.67 mg kg^−1^, CV = 172%). Arid and highlands both had the lowest B-exch (both at 0.07 g kg^−1^, CV = 86%).

Although no consistency of production systems association with the highest or lowest values for all soil proprieties, rainfed upland was associated with the lowest values of most soil properties: CEC (6.6 cmol(+) kg^−1^, CV = 61%), Clay (19.65%; CV = 60%), K-exch (0.08 g kg^−1^, CV = 50%), Ca-exch (0.59 g kg^−1^, CV = 81%), Mg-exch (0.13 g kg^−1^, CV = 113%), Exch-Cations (4.42 cmol(+) kg^−1^, CV = 80%), Cu-exch (1.67 mg kg^−1^, CV = 66%). Soil pH (5.37, CV = 14%) and B-exch (0.06 g kg^−1^, CV = 104%) were lowest for rainfed lowland; while TOC (1.6%, CV = 76%) and Bray-P (2.71 mg kg^−1^, CV = 133%) and Al-exch (6.78 g kg^−1^, CV = 35%) for irrigated lowland. Mn-exch was equally low for rainfed lowland and rainfed upland (0.06 mg kg^−1^, CV = 114% and 111%), and N for irrigated lowland and rainfed upland (0.14%, CV = 79% and 83%).

#### 3.1.3 Mineral fertilizer utilization rates

Average values of mineral fertilizer rates are found in Table 5 and coefficients of variation are shown in Table B3. The overall average values for N, P and K rates presented very large variations with CV of 147% for N fertilizer and 178% for both P and K fertilizers.

**Table 5:** Recorded mineral N, P, and K fertilizers application rates and grain yields of this study of “n” farmers’ fields for each country, across agro-ecological zones (AEZ) and production systems, and overall. Values are means of “n", and coefficients of variation (%) are between brackets for the overall data and are in supplementary information when not shown. Mineral fertilizer rates- (for each element) and grain yield values are displayed in colors ranging from green (lowest) to red (highest).

^a^n denotes the number of farmer’s fields

Across countries, application rates of N fertilizer in Senegal and Niger were largely above those of the rest of the studied countries (117 and 115 kg N ha^−1^, respectively with CV of 43% and 49%). The other countries excluding these later with Benin, Cameroon, Ghana, Mali, and Rwanda had very low N application rates ranging from zero to 16 kg N ha^−1^. Mineral P application rates were largest and above 20 kg P ha^−1^ for Ghana, Niger, and Rwanda. Very low to no application of P (0–9.5 kg P ha^−1^) was found for Burkina Faso, Cameroon, Chad, Côte d′Ivoire, DRC, Ethiopia, Guinea, Madagascar, Nigeria, Sierra Leone, Tanzania, The Gambia, and Uganda. For K fertilizer, rates were highest (19.8 – 30.3 kg K ha^−1^) for Benin, Ghana, Niger, and Rwanda. In return, the rest of the countries use very small (<12 kg K ha^−1^) to no K fertilizer.

Humid and sub-humid were associated with the lowest rates of the three fertilizers: ∼20 kg N ha^−1^,∼7.5 kg P ha^−1^ (CV = 141%), and ∼2 kg K ha^−1^ with large CV > 150%. Arid had the largest N, P, and K fertilizers application rates with 60 kg N ha^−1^ (CV = 99%), 16 kg P ha^−1^ (CV = 141%), and 12 kg K ha^−1^ (CV = 123%).

The application rates for all three mineral fertilizers were highest in irrigated lowland, followed by rainfed lowland and lowest in rainfed upland. These highest values in irrigated lowland were 59.6 kg N ha^−1^ (CV = 94%), 16.8 kg P ha^−1^ (CV = 120%) and 15.1 kg K ha^−1^ (CV = 116%), respectively; while 11.1 kg N ha^−1^ (CV = 220%), 5.4 kg P ha^−1^ (CV = 184%), and 5.7 kg K ha^−1^ (CV = 196%) for the lowest rates in rainfed upland.

### 3.2 Best models and prediction accuracy

For this section, only the selected models are shown in Table 6 but the results for all the models used are presented as supplementary information in Tables C1, C2, and C3. In this section, *R*^*2*^ cond. values refer to Nakagawa’s conditional coefficient.

**Table 6:** Selected best models for predicting N, P, K, Ca, Mg, S, Fe, B, Mn, and Cu concentrations in rice grain in this study.

Nutrients	Selected model IDs	Model composition	AIC^a^	*R*^*2*^ cond.^b^	*R*^*2*^ marg.^b^	Decomposed *R*^*2*^ marg.^c^	EF^d^
**N**	Mod N1	AEZ	-519	0.30	0.11	0.11	0.219
**P**	Mod P2	AEZ+Production systems	-3455	0.61	0.25	0.17 – 0.08	0.430
**K**	Mod K6	AEZ+Production systems+Exch-Cations	-2841	0.74	0.20	0.14 – 0.06 – < 0.01	0.527
**Ca**	Mod Ca1	AEZ	-7127	0.27	0.07	0.07	0.178
**Mg**	Mod Mg2	AEZ+Production systems	-5051	0.67	0.25	0.09 – 0.17	0.477
**S**	Mod S1	AEZ	-5963	0.50	0.16	0.16	0.380
**Fe**	Mod Fe8	AEZ+Production systems+N rate+P rate+K rate	13139	0.52	0.22	0.16 – < 0.01 – 0.05	0.370
**B**	Mod B6	AEZ+Production systems+Exch-Cations	942	0.79	0.19	0.15 – 0.02 – 0.01	0.584
**Mn**	Mod Mn4	AEZ+Production systems+TOC	8285	0.48	0.04	0.05 – < 0.00 – < 0.00	0.414
**Cu**	Mod Cu4	AEZ+Production systems+TOC	7138	0.37	0.11	0.09 – < 0.00 – 0.02	0.256

a Akaike Information Criteria, more negative AIC equals a greater relative quality compared to other models for a set of data. ^b^Nakagawa’s conditional and marginal coefficients of determination. ^c^Marginal *R*^*2*^ decomposed for each fixed-factor variable of the selected models. ^d^Modeling efficiency: positive values indicate predicted concentrations with the models are better than average concentration values with EF equal to 1 being a perfect model

Models with AEZ as the only predictor variable (Mod N1, Mod Ca1, and Mod S1) performed best (lowest AIC) for N, Ca, and S concentrations in rice grain, with *R*^*2*^ cond. values of 0.3, 0.27, and 0.50, respectively. There was a small improvement in *R*^*2*^ cond. values for grain N and Ca concentrations when all soil parameters were added together in one model (Mod N3 and Mod Ca3 in tables C1 and C2) but these models were not selected because of parsimony (higher AIC). For P and Mg concentrations, models Mod P2 and Mod Mg2 with AEZ and production systems as predictor variables were the best models among the largest *R*^*2*^ cond. values of 0.61 and 0.67, respectively. K and B concentrations were both best explained by AEZ, production systems, and Exch-Cations and their *R*^*2*^ values reached 0.74 and 0.79. A small improvement of the *R*^*2*^ cond. value was also obtained for B concentration when all soil parameters were used in one model but not selected because the improvement in *R*^*2*^ cond. is small (0.015, see Table C3). Models Mod Mn4 (*R*^*2*^ cond. = 0.49) and Mod Cu4 (*R*^*2*^ cond. = 0.38) with TOC as an additional predictor variable were selected for Mn and Cu concentrations despite their slightly larger AIC than the model with all soil parameters (Mod Mn3 and Mod Cu3). Lastly, model Mod Fe8 with N, P, and K rates as additional variables was selected over Mod Fe3 as there was only 0.05 improvement in *R*^*2*^ cond. occurred (Table C2) for Fe concentration. The *R*^*2*^ cond. value for this Mod F8 was 0.52.

Modeling efficiency (EF) was used to test the prediction accuracy of the above-selected models. For all the 10 nutrients, EF was positive meaning that our selected models are better predictors than only using the average values of these nutrients in rice grain (Table 6). Models Mod K6 and Mod B6 had high EF (>0.5), followed by Mod Mg2, Mod P2, and Mod Mn4 (0.41 – 0.48). Then, models Mod S1 and Mod Fe8 with 0.38 and 0.37 of EF respectively. Finally, the EF of the selected models for N, Ca, and Cu concentrations were below 0.260.

### 3.3 Predictor variables’ importance for the best models

The importance of predictor variables can also be assessed from *R*^*2*^ cond. and marginal *R*^*2*^ (*R*^*2*^ marg.) in Table 6. First, differences between *R*^*2*^ cond. and marginal *R*^*2*^ marg. for all 10 nutrients (details not shown) show that the amount of variance accounted by the variables used as fixed factors is smaller than the variance explained by “country” which is used as a random factor. In other words, such suggests that “country” would have more importance than the other variables used as fixed factors in the models for the estimation of the nutrient concentrations in rice grain for our study.

With regard to the fixed-factor variables, AEZ appears to be more influential for all the nutrients i.e. largest *R*^*2*^ marg. in Table 6 (see column Decomposed *R*^*2*^ marg.) except for Mg concentration where production systems had the highest value of 0.17 against 0.09 for AEZ. Production systems had a slightly large *R*^*2*^ marg. for K and B concentrations than Exch-Cations. In opposition, production systems had a smaller *R*^*2*^ marg. than N, P, and K rates for Fe concentration and than TOC for Cu concentration.

### 3.4 Coefficients of variable estimates for the best models

Model coefficients for the fixed factors were generated to quantify the extent of influence of each level of dependent variables within the best models for all 10 nutrients. In Table 7, coefficients of sub-humid for models explained by AEZ only, and of sub-humid and irrigated lowland for models explained by AEZ and production systems are shown as references together with the increase/decrease due to the other variables from these reference coefficients.

**Table 7:** Coefficients of variable estimates of fixed factors from the selected prediction models in Table 6.

Variables	Mod N1	Mod P2	Mod K6	Mod Ca1	Mod Mg2	Mod S1	Mod Fe8	Mod B6	Mod Mn4	Mod Cu4
Intercept^a^	1.43 * **	0.259 * **	0.373 * **	0.043 * **	0.125 * **	0.097 * **	145 * **	2.28 * **	58.6 * **	18.1 * **
AEZ/Arid	0.06 *	-0.012 ns	-0.073 * **	0.001 ns	-0.024 * **	0.009 * **	146 * **	-0.65 * **	1.81 ns	7.17 * **
AEZ/Highlands	-0.13 * **	0.003 ns	0.016 ns	0.004 * *	0.01 *	-0.008 * *	318 * **	0.12 ns	6.26 ns	5.36 * *
AEZ/Humid	0.1 * *	0.09 * **	0.078 * **	-0.003 *	0.015 * **	0.012 * **	127 * *	0.22 *	-1.54 ns	2.82 ns
Production systems/rainfed lowland		-0.014 * *	-0.02 *		-0.007 * *		-21 ns	-0.11 *	1.19 ns	0.87 *
Production systems /rainfed upland		-0.047 * **	-0.059 * **		-0.024 * **		-74 * **	-0.31 * **	5.48 * *	-2.02 * **
N rate							-0.8 * **			
P rate							0.4 ns			
K rate							0.4 ns			
Exch-Cations			-0.002 * **					-0.02 * **		
TOC									1.55 * **	-0.95 * **

a Refers to categorical variables AEZ/Sub-humid for models explained by AEZ only, and to AEZ/Sub-humid and production systems/irrigated lowland for models explained by AEZ and production systems. Values for the other variables represent an increase/decrease from the intercept. Values in bold and followed by asterisks are significantly different from the intercept with * ** *p* < 0.0001, * * *p* < 0.001, * *p* < 0.05

Among AEZ, humid had the largest positive significant impact on N, P, K, Mg, S, and B concentrations in rice grain over the reference sub-humid. For Ca and Fe concentrations, significantly highest positive coefficients were obtained for highlands, particularly high for Fe concentration. For Mn concentration, sub-humid had the largest effect, and arid for Cu concentration.

When production systems were included in the best models, irrigated lowland was always the production system with the largest coefficients with the exception of Mn and Cu where rainfed upland and rainfed lowland had the largest ones, respectively. rainfed upland tended to have the smallest ones in the estimation of P, K, Mg, Fe, B, and Cu in rice grain in the opposite of Mn concentration which was found for rainfed lowland and irrigated lowland.

With N, P, and K rates retained for the prediction of Fe concentration in model Mod Fe8, only N rate was significant and it decreased the concentration of Fe in rice grain.

In model Mod K6, Exch-Cations contributed negatively with the concentration of K in grain although with a relatively small effect (coefficient at least 10 times smaller than for the other variables – factors). A similar trend is also observed for model Mod B6.

Both prediction models for Mn and Cu concentrations (Mod Mn4 and Mod Cu4) included TOC but the first was positively affected by it while it had a negative effect on the second.

### 3.5 Relationship between estimation-based versus average-based nutrient uptakes with actual nutrient uptakes

**Fig. 3:**
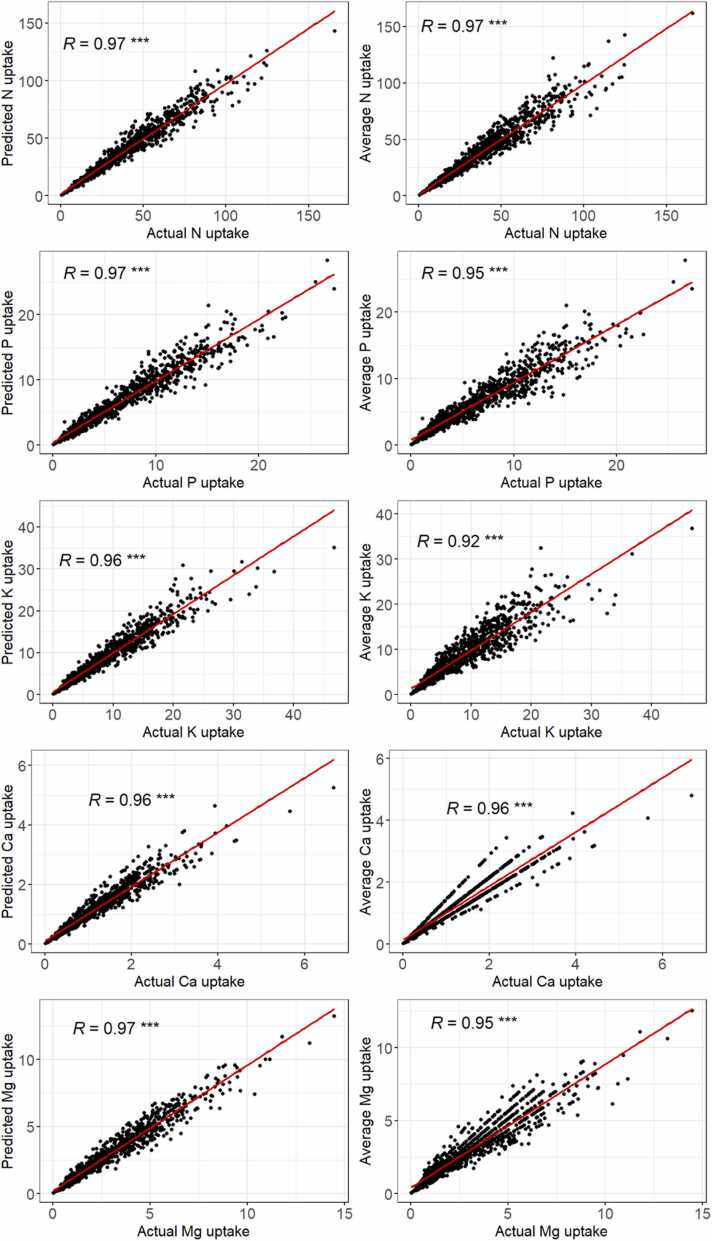
Relationship between actual N, P, K, Ca, and Mg uptakes and predicted uptakes (left) from best models against average uptakes (right) using Pearson’s correlation test (significance level set at *p* < 0.05). * ** *p* < 0.001.

**Fig. 4:**
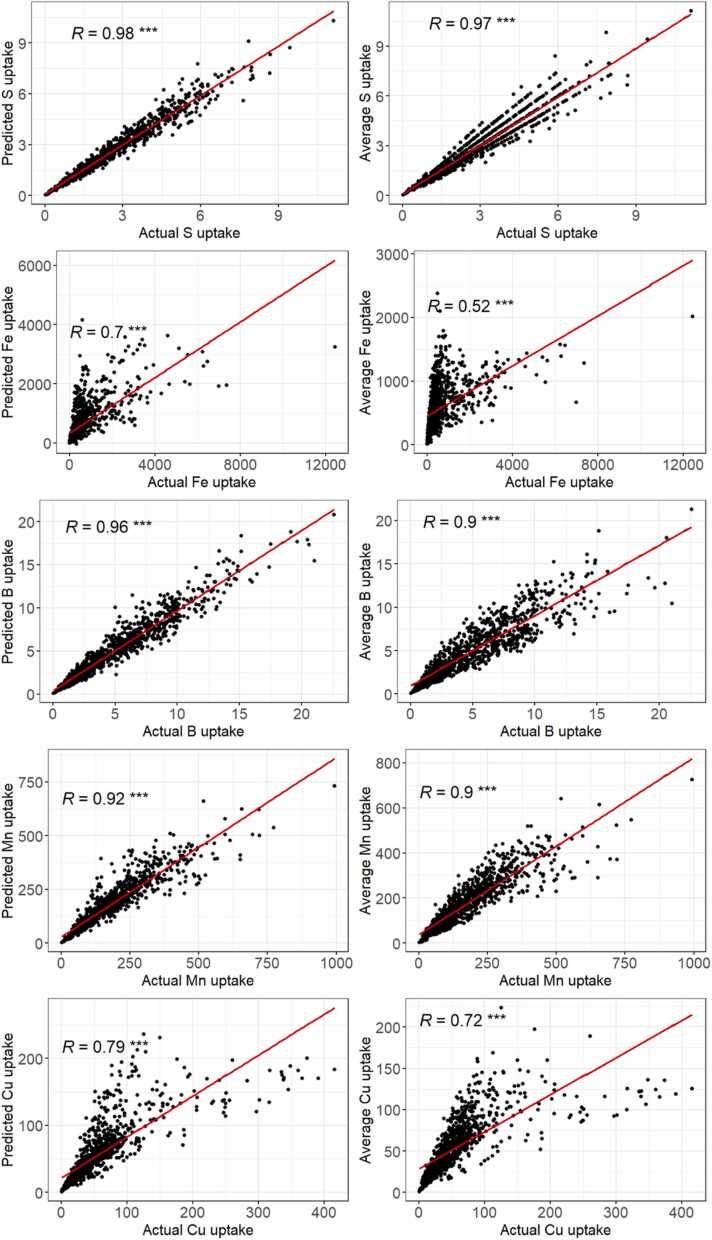
Relationship between actual S, Fe, B, Mn, and Cu uptakes and predicted uptakes (left) from best models against average uptakes (right) using Pearson’s correlation test (significance level set at *p* < 0.05). * ** *p* < 0.001.

Prediction accuracy was evaluated with the relationships between predictions of nutrient uptake of selected models with actual nutrient uptake against average nutrient uptake with actual nutrient uptake (Fig. 3, Fig. 4).

Given that nutrient uptakes are calculated on the basis of grain yield, summary data of this latter are presented in Table 5. Grain yields varied largely for each country, across AEZ and production systems as well with an overall average value of 2.7 t ha^−1^ (CV = 64%). Niger (5.3 t ha^−1^, CV = 18%) and Cameroon (4.1 t ha^−1^, CV = 62%) registered the highest yields. Across production systems, highest grain yield was observed in highlands reaching 3.8 t ha^−1^ (CV = 53%) and lowest in sub-humid (2 t ha^−1^, CV = 63%). Obviously, irrigated lowland was associated with the highest yield across AEZ with 3.7 t ha^−1^ (CV = 41) while the lowest yield was observed for rainfed upland (1.4 t ha^−1^, CV = 60%).

Nutrient uptakes predicted with the selected models were in all cases better than the uptakes calculated from average values but the differences in correlation coefficients *R* were rather small (<0.05) except for Fe, B, and Cu uptakes where the difference in *R* was superior to 0.05. However, prediction accuracy assessed with EF (see Table 6) suggests that only the model Mod B6 for predicting the concentration of B in rice grain is satisfactory (EF = 0.584) and is proposed to be used to estimate B uptake by rice grain.

## 4 Discussion

### 4.1 Estimation of macro and micronutrient concentrations and uptakes in rice grain in SSA

Fast but reliable quantification of nutrient concentrations in rice grain is needed towards sufficient and nutritionally superior quality rice production with less environmental footprints. The overall objective of this study is to provide simple approaches for the estimation of N, P, K, Ca, Mg, S, and Fe, Mn, B, Cu concentrations in rice grain in SSA with a limited set of variables within categories of AEZ, production systems, soil properties and mineral fertilizer use.

This is the first study dealing with rice grain nutrient estimation for SSA and we succeeded to develop parsimonious predictive mixed-effect models for estimating all these 10 nutrient concentrations (Table 6). With Nakagawa’s conditional *R*^*2*^ ranging from 0.28 for Ca to 0.76 for B, our selected best models allow reasonable estimation of all investigated nutrient concentrations in this study. Additionally, prediction accuracy tested with modeling efficiency (EF) metric showed that all best models allow better estimation (EF>0) than just average concentrations. However, without taking into account nutrient uptakes, only the estimation of concentrations of K and B had high EF larger than 0.5 (0.527 and 0.584, respectively) which allows considering these models as satisfactory (Moriasi et al., 2007).

In relation to the estimation of nutrient removal which is derived from nutrient uptakes, comparisons of relationships between estimation-based nutrient uptakes with actual nutrient uptakes and relationships between average-based with actual nutrient uptakes suggest only the estimation of B uptake in rice grain was improved by using our best models (Fig. 3). Uptakes of the other nutrients in rice grains can be simply estimated using average concentration values at the regional level for SSA. Our result could be supported by previous studies, suggesting that nutrient uptakes or removals could be more driven by crop yield rather than nutrient concentrations (Jones et al., 2013, Kekulandara et al., 2019, McGrath, 1985). Average nutrient concentrations at the regional scale for use in estimates of crop nutrient uptake make for a simpler approach than the modeling approach used in this study. However, the disadvantage of using average values is that it relies on (expensive and time-consuming) surveys to get these values in the future, as some of the explanatory variables could be changed over time (e.g. fertilizer application rate). Further research is needed for improving model accuracy.

In this study, we adapted a methodology developed by Ludemann et al. (2022) which was tested on maize and proposed to be applied to other crops. Although Ludemann et al. (2022) found that machine learning such as Random Forests tends to outperform the linear mixed-effects regression when predicting N concentration in maize grain, we opted for the latter because it allows better interpretation of the results given that one of our objectives was to identify the most influencing factors of nutrient concentrations in rice grain in SSA together with the underlying mechanisms. Further studies are suggested to improve the models by testing different machine learning algorithms such as Random Forests, Support Vector Machine or Gradient Boosting. Our study provides additional non-explored aspects to the methodology in terms of predictor variables namely AEZ and production systems. One limitation of our models would be the omission of rice variety (genotype) which is an important factor that can be influential for the estimation of nutrient concentrations in cereal crops. Practically, surveyed farmers in SSA use too many different rice varieties across and within sites, and to include variety as a predictor variable in the mixed-effect models would complicate results interpretation within the scope of this study. Previous studies observed significant variations of nutrient concentrations in rice grains having a genetic basis (Johnson et al., 2021, Pinson et al., 2015). For example, concentrations of macronutrients (N, P, K, Ca, and Mg) in rice grains evaluated on very large accessions of 529 cultivated rice displayed genotypic variations ranging from 10% to 38%, while those of micronutrients (Fe, Cu, Mn, and B) had 20–191% variations (Yang et al., 2018) and up to 140% for S on 1763 accessions (Pinson et al., 2015). For the particular case of P, Vandamme et al. (2016) observed large genotypic variations of its concentration in rice grain including those cultivated in Africa. Evaluation of nutrient concentrations of popular rice varieties in SSA through growing these varieties in the same field is needed.

### 4.2 Relationships between predictor variables and nutrient concentrations in predictor models

Overall, this study found that the variable country has the most contribution to the variations of concentrations in rice grain of the 10 investigated nutrients for SSA in our best models. Such adds to previous observations of Johnson et al. (2021) where AEZ and production systems were found to be most influential. Although clear differences between the different countries are observable in terms of soil properties and mineral fertilizer application rates (Table 4, Table 5), these factors subtly or did not contribute to the best models. Such implies that other factors such as the timing of fertilizer applications (Pan et al., 2012, Shi et al., 1995), rice variety as cited above, occurrence of drought periods (Mumtaz et, 2020; ben Mariem et al., 2021), atmospheric CO_2_ concentration (ben Mariem et al., 2021, Zhu et al., 2018) should be investigated as a potential improvement of our best models.

Coming after the variable country, AEZ dominates the variations of concentrations of most of the investigated nutrients in rice grain in the region. Du et al. (2013) found that environment and climate, in particular, may be key factors affecting elemental accumulation in plants by modifying plant growth and metabolism. Temperature and humidity are likely the most influential climate factors (Liu et al., 2017, Wang et al., 2018). However, it was not expected that specific effects of AEZ showed no consistency between nutrients (Table 3, Table 7), and the state of current knowledge limits our capacity in explaining such. Nevertheless, our findings are consistent with those of Gashu et al. (2021) where stronger geographical effects than agronomical interventions on micronutrient concentrations in crop grain including rice in Ethiopia and Malawi were observed.

Oppositely, production systems effects were consistent with irrigated lowland having the largest values for all nutrients except for Mn and Cu concentrations for which rainfed upland and rainfed lowland were highest (Table 7). This is in accordance with the general fertility advantages of waterlogged systems of irrigated lowland and rainfed lowland over the non-waterlogged system of rainfed upland with ameliorated chemical fertility, accumulation of organic matter, and improved availability of some macro and micronutrients (Sahrawat, 2012). Physicochemical changes induced by soil waterlogging favor the availability of some nutrients which consequently affects their accumulation in rice grains: NH_4_-N, K, Ca, and Mg (Sahrawat, 2000), P (Rakotoson et al., 2022, Sahrawat, 2000), Fe (Sahrawat, 2012), and possibly B (Neue and Mamaril, 1984). The larger positive effect of rainfed upland on Mn concentration is in theory opposing thermodynamics prediction of reduction sequence in waterlogged soil which should increase its availability (Kirk, 2004, Ponnamperuma, 1972), but Sahrawat (2012) reported the opposite. Although rainfed lowland was higher than irrigated lowland for Cu concentration, these two are lowland systems and are subject to soil waterlogging which is also known to increase Cu availability (Mitchell, 1964). Additionally, waterlogged systems are in general more productive as it is less prone to water stress and less affected by weed infestation (Ponnamperuma, 1975) which can impact rice root access to soil plant nutrients. Another important contributing factor could also be the high rates of mineral N, P, and K fertilizers applied in irrigated lowland fields compared to the other two production systems in this study which confirms previous observations (Dossou-Yovo et al., 2020, Niang et al., 2017).

Soil Exch-Cations, TOC, and NPK application rates add to AEZ and production systems (besides country as a random factor) for the estimation of K and B, Mn and Cu, and Fe concentrations in rice grain, respectively. For K, a negative coefficient (Table 7) of Exch-Cations is remarkably against all expectations, and we assume that its effect is nested within that of the other predictor variables which are largely bigger (∼10 times). Such could be also the case for B concentration. The large positive effect of TOC on Mn concentration can be caused by the promoted formation of organic chelating agents which can enhance its availability for plant roots i.e preventing its fixation by inorganic compounds or from precipitations (Weil and Brady, 2017). The same concept of chelation applies to Cu concentration but in return forms water-insoluble complexes explaining the negative effect of TOC (Weil and Brady, 2017). The same authors reported poor drainage and high organic matter in soil can lead to Cu deficiency for crops. Last, some studies have not reported any effect of fertilizer use on Fe accumulation in rice grain (Chandel et al., 2010, Rakotoson et al., 2019), which is somehow in line with the non-significant coefficients of P and K application rates in our study. However, we found that N application rates were negatively affecting Fe accumulation and such can be because of the high rates (∼200 kg N ha^−1^) considered as possibly excessive (Panda et al., 2012, Su et al., 2018). The implication of this on Fe fortification of rice grain would be about the balance of N fertilization rates with yields.

### 4.3 Future priority setting

Optimal values (calculated based on Dobermann and Fairhurst, 2000) in Table 3 can be used as references and therefore to identify which countries, but also which AEZ and production systems are to be targeted for future interventions. Since Mn and Cu concentrations in the rice grain of this study are all far above the optimal values, with Ca and S concentrations being around optimal values of 0.05% and 0.1%, respectively, future efforts should be shifted towards the other nutrients.

Concentrations of K, Mg, and B were all below optimal values for all 20 countries and should be a priority target nutrient for future interventions, including the relationship with grain yields. Probable causes of low K in rice grain in SSA countries are negative K balance described in Majumdar et al. (2021). It is rather surprising to see sub-optimal Mg concentrations even in irrigated lowland which should not suffer from Mg deficiency because adequate amounts are usually supplied in irrigation water (Dobermann and Fairhurst, 2000). Low values for rainfed systems (rainfed lowland and rainfed upland) could be caused by depletion of soil Mg due to continuous removal of Mg without recycling of crop residues or replacement with mineral fertilizer (Dobermann and Fairhurst, 2000). For B, sub-optimal values could be just because of low soil available B (Dobermann and Fairhurst, 2000) and no B fertilizer use (Atique-ur-Rehman et al., 2018).

Beside K, Mg, and B, needless to mention the importance of Fe concentrations with reference to rice fortification. Despite an overall average slightly above optimal for this element, results clearly identified locations (countries) where values were very low, but also below optimal in humid and sub-humid acro-ecologies and under rainfed upland production system. Half of the countries in this study had sub-optimal Fe concentrations in rice grains (Table 3). Why grains of rice of rainfed upland are low in Fe is already known and explained above. Graham et al. (1999) observed significant environmental effects which additionally interacted with genotypes confirming the importance of inter-country, AEZ, and production systems variations.

For the rest of the nutrients that are N and P, their overall average values were both slightly below optimal but also displayed inter-country, AEZ, and production systems variations. For both of them, half of the countries also had below-optimal values (Table 3). It has been confirmed earlier that N and P are common limiting nutrients in rice production in SSA (Saito et al., 2019).

## 5 Conclusion

Our main objectives were to examine if the concentrations of macro- (N, P, K, Ca, Mg, S) and micronutrients (Fe, Mn, B, Cu) in rice grain can be estimated using AEZ, production systems, soil properties, and mineral fertilizer N, P, and K application rates, and to identify if nutrient uptakes estimated by best-fitted models with above variables provide a better prediction of actual nutrient uptakes than average-based uptakes in SSA. Our study was successful in generating mixed-effect predictive models that can reasonably estimate concentrations of all 10 nutrients in rice grain. However, only the estimations of K and B concentrations were satisfactory (modeling efficiency EF > 0.5). The variable country rather than AEZ and production systems most contributed to the variation of concentrations of these nutrients in our best predictive models. Despite greater positive relationships between actual nutrient uptakes and model estimation-based uptakes than those between actual nutrient uptakes and average-based uptakes, the estimation of only B uptake was significantly improved. Our findings suggest that estimates of the macronutrient and micronutrient uptakes in rice grain can be obtained simply by using average concentrations of each nutrient at the regional scale for SSA with the exception of B associated with high modeling efficiency EF and an improved uptake over the average-based uptake. Additionally, further investigation of other country-specific factors such as the timing of fertilizer applications, rice variety, occurrence of drought periods, and atmospheric CO_2_ concentration is proposed as a potential improvement of our best predictive models and so for future studies on a smaller scale.

## Funding sources

This study was financially supported by the 10.13039/501100000780European Union and 10.13039/100008687International Fund for Agricultural Development (IFAD) under the project “Sustainable and Diversified Rice-based Farming Systems [DCIFOOD/2015/360-968]” under the program “Putting Research into Use for Nutrition, Sustainable Agriculture and Resilience (PRUNSAR)”, and the Bill & Melinda Gates Foundation (BMGF), Seattle, USA, Grant ID INV-005431 through the 10.13039/501100015815CGIAR Excellence in Agronomy 2030 (Incubation Phase).

## Declaration of Competing Interest

The authors declare the following financial interests/personal relationships which may be considered as potential competing interests:Africa Rice Center reports financial support was provided by European Union. Africa Rice Center reports financial support was provided by International Fund for Agricultural Development. Africa Rice Center reports financial support was provided by Bill & Melinda Gates Foundation.

## Data Availability

The data will be made available in an open repository: Africa Rice Dataverse.
