# Supplementary material for: Estimating nutrient concentrations and uptake in rice grain in sub-Saharan Africa using linear mixed-effects regression
Source: Field Crops Res. 2023 Aug 1;299:108987. doi: 10.1016/j.fcr.2023.108987 (PMC10300240; doi:10.1016/j.fcr.2023.108987)
Supplement: Supplementary file 1 — Supplementary material. [file mmc1.docx]

**Appendix A. Summary statistics of response and predictor variables (n = 998)**

|  | **Mean** | **SD** | **Median** | **Min.** | **Max.** | **Skewness** | **Kurtosis** | **IQR** | **Q0.25** | **Q0.75** |
| --- | --- | --- | --- | --- | --- | --- | --- | --- | --- | --- |
| **Soil properties** | | | | | | | | | | |
| pH | 5.51 | 0.71 | 5.5 | 3.3 | 7.9 | 0.24 | 0.31 | 1 | 5 | 6 |
| N (%) | 0.16 | 0.14 | 0.11 | 0.02 | 1.05 | 2.32 | 7.18 | 0.13 | 0.07 | 0.2 |
| TOC (%) | 1.77 | 1.54 | 1.29 | 0.27 | 10.89 | 2.34 | 7.31 | 1.44 | 0.74 | 2.18 |
| Bray-P (mg kg^-1^) | 3.65 | 4.76 | 2.08 | 0.03 | 44.7 | 3.58 | 17.88 | 2.98 | 1.12 | 4.1 |
| Clay (%) | 28.65 | 18.48 | 25 | 2 | 91 | 0.94 | 0.54 | 26 | 14 | 40 |
| K-exch (g kg^-1^) | 0.09 | 0.05 | 0.08 | 0.03 | 0.47 | 1.66 | 4.35 | 0.06 | 0.06 | 0.11 |
| Ca-exch (g kg^-1^) | 1.06 | 1.12 | 0.66 | 0.08 | 8.57 | 2.38 | 6.16 | 0.79 | 0.39 | 1.17 |
| Mg-exch (g kg^-1^) | 0.27 | 0.35 | 0.14 | 0.02 | 3.08 | 2.91 | 10.62 | 0.2 | 0.08 | 0.28 |
| Mn-exch (mg kg^-1^) | 0.07 | 0.07 | 0.05 | 0 | 0.52 | 2.4 | 8.25 | 0.06 | 0.03 | 0.09 |
| Exch-Cations cmol(+) kg^-1^ | 8.21 | 8.76 | 5.13 | 0.91 | 68.6 | 2.45 | 6.74 | 6.12 | 3.07 | 9.2 |
| Cu-exch (mg kg^-1^) | 2.16 | 1.29 | 1.75 | 0.5 | 11.36 | 1.77 | 4.36 | 1.34 | 1.28 | 2.62 |
| B-exch (mg kg^-1^) | 0.08 | 0.09 | 0.05 | 0.01 | 0.97 | 3.38 | 18.54 | 0.07 | 0.03 | 0.1 |
| Al-exch (g kg^-1^) | 7.93 | 4.62 | 6.96 | 1.92 | 28.57 | 1.46 | 2.31 | 5.38 | 4.42 | 9.8 |
| CEC cmol(+) kg^-1^ | 11.76 | 10.01 | 8.6 | 1.99 | 64.13 | 2.12 | 4.61 | 8.2 | 5.33 | 13.54 |
|  |  |  |  |  |  |  |  |  |  |  |
| **Mineral fertilizer rates (kg ha^-1^)** | | | | | | | | | | |
| N | 35.23 | 51.84 | 1.6 | 0 | 200 | 1.65 | 2 | 57.5 | 0 | 57.5 |
| P | 10.35 | 18.39 | 0 | 0 | 200 | 3.77 | 26.59 | 18.59 | 0 | 18.59 |
| K | 9.13 | 16.27 | 0 | 0 | 200 | 3.36 | 23.16 | 15.76 | 0 | 15.76 |
|  |  |  |  |  |  |  |  |  |  |  |
| **Grain Yield**  **(t ha^-1^)** | 2.69 | 1.73 | 2.38 | 0.03 | 11.13 | 0.87 | 0.64 | 2.46 | 1.27 | 3.73 |
|  |  |  |  |  |  |  |  |  |  |  |
| **Nutrient concentration in rice grain** | | | | | | | | | | |
| N (%) | 1.45 | 0.2 | 1.44 | 0.91 | 2.32 | 0.41 | 0.22 | 0.27 | 1.31 | 1.58 |
| P (%) | 0.25 | 0.05 | 0.25 | 0.07 | 0.49 | 0.57 | 0.98 | 0.07 | 0.21 | 0.28 |
| K (%) | 0.33 | 0.08 | 0.32 | 0.16 | 0.6 | 0.71 | 0.41 | 0.09 | 0.28 | 0.37 |
| Ca (%) | 0.04 | 0.01 | 0.04 | 0.03 | 0.07 | 0.41 | 0.41 | 0.01 | 0.04 | 0.05 |
| Mg (%) | 0.11 | 0.02 | 0.11 | 0.05 | 0.21 | 0.42 | 0.29 | 0.03 | 0.1 | 0.13 |
| S (%) | 0.1 | 0.01 | 0.1 | 0.06 | 0.16 | 0.36 | 0.25 | 0.02 | 0.09 | 0.11 |
| Fe (mg kg^-1^) | 213 | 214 | 133 | 32.4 | 2232 | 2.99 | 14.11 | 159 | 88.3 | 247 |
| Mn (mg kg^-1^) | 65.11 | 19.25 | 61.99 | 22.89 | 146 | 0.86 | 0.82 | 24.51 | 51.13 | 75.65 |
| B (mg kg^-1^) | 1.91 | 0.55 | 1.85 | 0.79 | 3.86 | 0.41 | -0.38 | 0.79 | 1.5 | 2.29 |
| Cu (mg kg^-1^) | 20.05 | 9.64 | 17.78 | 6.33 | 71.25 | 2.35 | 6.79 | 8.87 | 14.11 | 22.98 |

**Appendix B. Coefficients of variation of response and predictor variables**

**Table B1.** Coefficients of variation (%) values of N, P, K, Ca, Mg, S, Fe, B, Mn, and Cu concentrations in rice grain farmers’ fields for each country, and across agro-ecological zones (AEZ) and production systems (PS), and overall.

| **Country/AEZ/PS^a^** | **n^b^** | **N** | **P** | **K** | **Ca** | **Mg** | **S** | **Fe** | **B** | **Mn** | **Cu** |
| --- | --- | --- | --- | --- | --- | --- | --- | --- | --- | --- | --- |
| **Country** |  |  |  |  |  |  |  |  |  |  |  |
| Benin | 105 | 12 | 14 | 16 | 15 | 16 | 13 | 92 | 18 | 21 | 63 |
| Burkina Faso | 11 | 14 | 9 | 12 | 11 | 13 | 13 | 31 | 17 | 12 | 18 |
| Cameroon | 74 | 11 | 14 | 18 | 16 | 13 | 13 | 134 | 15 | 21 | 27 |
| Chad | 9 | 4 | 12 | 8 | 11 | 6 | 6 | 32 | 7 | 14 | 11 |
| Côte d’Ivoire | 69 | 14 | 17 | 19 | 14 | 16 | 13 | 44 | 25 | 22 | 49 |
| DRC | 24 | 9 | 8 | 16 | 15 | 15 | 9 | 26 | 18 | 18 | 23 |
| Ethiopia | 23 | 9 | 14 | 14 | 14 | 18 | 9 | 33 | 13 | 13 | 28 |
| Ghana | 97 | 13 | 21 | 21 | 14 | 16 | 12 | 56 | 24 | 19 | 25 |
| Guinea | 56 | 15 | 17 | 13 | 18 | 15 | 14 | 109 | 22 | 18 | 58 |
| Madagascar | 54 | 10 | 16 | 15 | 15 | 17 | 10 | 45 | 12 | 18 | 15 |
| Mali | 65 | 14 | 28 | 15 | 16 | 30 | 11 | 85 | 27 | 24 | 15 |
| Niger | 14 | 14 | 13 | 10 | 15 | 10 | 11 | 25 | 10 | 13 | 16 |
| Nigeria | 22 | 8 | 8 | 10 | 13 | 10 | 8 | 37 | 13 | 19 | 11 |
| Rwanda | 45 | 12 | 18 | 11 | 22 | 13 | 9 | 48 | 10 | 42 | 68 |
| Senegal | 36 | 11 | 11 | 15 | 16 | 11 | 11 | 25 | 13 | 13 | 16 |
| Sierra Leone | 74 | 13 | 14 | 14 | 12 | 14 | 15 | 37 | 21 | 18 | 31 |
| Tanzania | 57 | 17 | 17 | 16 | 14 | 17 | 11 | 83 | 24 | 36 | 27 |
| The Gambia | 106 | 11 | 21 | 23 | 15 | 22 | 9 | 75 | 23 | 21 | 26 |
| Togo | 42 | 9 | 10 | 19 | 12 | 14 | 12 | 54 | 26 | 10 | 14 |
| Uganda | 15 | 15 | 15 | 16 | 14 | 11 | 14 | 27 | 11 | 19 | 15 |
|  |  |  |  |  |  |  |  |  |  |  |  |
| **AEZ** |  |  |  |  |  |  |  |  |  |  |  |
| Arid | 300 | 13 | 21 | 23 | 16 | 23 | 13 | 91 | 26 | 24 | 53 |
| Highlands | 169 | 13 | 16 | 22 | 18 | 17 | 14 | 87 | 24 | 35 | 46 |
| Humid | 106 | 14 | 23 | 25 | 14 | 20 | 13 | 64 | 31 | 22 | 42 |
| Sub-humid | 423 | 13 | 21 | 21 | 16 | 21 | 15 | 83 | 31 | 32 | 42 |
|  |  |  |  |  |  |  |  |  |  |  |  |
| **PS** |  |  |  |  |  |  |  |  |  |  |  |
| IL | 326 | 14 | 18 | 21 | 17 | 17 | 14 | 104 | 27 | 30 | 59 |
| RL | 438 | 14 | 23 | 24 | 15 | 24 | 15 | 94 | 27 | 28 | 29 |
| RU | 234 | 14 | 18 | 23 | 16 | 22 | 155 | 88 | 33 | 31 | 53 |
|  |  |  |  |  |  |  |  |  |  |  |  |
| **Overall** | 998 | 14 | 21 | 23 | 16 | 22 | 14 | 100 | 30 | 29 | 48 |

**^a^**AEZ: 5 classes reduced to 4 for Sub-Saharan Africa (arid and semi-arid combined) (HarvestChoice and (IFPRI), 2015). *****IL=irrigated lowland, RL=rainfed lowland, RU=rainfed upland. ^b^n denotes the number of farmer’s fields

**Table B2.** Coefficients of variation (%) of the relevant soil properties for this study in farmers’ fields for each country, and agro-ecological zones (AEZ) and production systems (PS), and overall.

| **Country/AEZ/PS** | **n^b^** | **pH** | **CEC** | **TOC** | **Clay** | **N** | **Bray-P** | **K-exch** | **Ca-exch** | **Mg-exch** | **Mn-exch** | **Exch-Cations** | **Cu-exch** | **B-exch** | **Al-exch** |
| --- | --- | --- | --- | --- | --- | --- | --- | --- | --- | --- | --- | --- | --- | --- | --- |
| **Country** |  |  |  |  |  |  |  |  |  |  |  |  |  |  |  |
| Benin | 105 | 11 | 55 | 63 | 77 | 66 | 91 | 38 | 61 | 64 | 67 | 57 | 41 | 63 | 39 |
| Burkina Faso | 11 | 10 | 26 | 38 | 32 | 47 | 60 | 18 | 30 | 34 | 41 | 28 | 33 | 45 | 42 |
| Cameroon | 74 | 14 | 50 | 84 | 44 | 81 | 113 | 32 | 96 | 92 | 46 | 87 | 49 | 112 | 51 |
| Chad | 9 | 5 | 40 | 66 | 45 | 68 | 31 | 36 | 30 | 40 | 35 | 32 | 16 | 37 | 57 |
| Côte d’Ivoire | 69 | 11 | 38 | 79 | 37 | 71 | 83 | 42 | 50 | 53 | 74 | 48 | 39 | 153 | 41 |
| DRC | 24 | 16 | 87 | 41 | 28 | 36 | 89 | 45 | 148 | 151 | 209 | 135 | 83 | 131 | 22 |
| Ethiopia | 23 | 7 | 26 | 21 | 17 | 26 | 146 | 31 | 38 | 37 | 30 | 36 | 29 | 41 | 13 |
| Ghana | 97 | 9 | 77 | 64 | 74 | 65 | 156 | 57 | 79 | 88 | 69 | 81 | 63 | 89 | 40 |
| Guinea | 56 | 12 | 70 | 78 | 43 | 86 | 78 | 55 | 86 | 128 | 92 | 88 | 42 | 97 | 49 |
| Madagascar | 54 | 7 | 48 | 52 | 25 | 50 | 79 | 28 | 52 | 64 | 62 | 52 | 39 | 45 | 24 |
| Mali | 65 | 11 | 31 | 34 | 34 | 40 | 121 | 23 | 31 | 46 | 43 | 31 | 32 | 33 | 37 |
| Niger | 14 | 7 | 37 | 27 | 22 | 25 | 89 | 30 | 43 | 52 | 46 | 44 | 28 | 47 | 12 |
| Nigeria | 22 | 19 | 33 | 61 | 63 | 77 | 94 | 22 | 40 | 43 | 67 | 37 | 28 | 86 | 29 |
| Rwanda | 45 | 4 | 10 | 32 | 26 | 41 | 109 | 11 | 16 | 19 | 31 | 15 | 17 | 26 | 11 |
| Senegal | 36 | 7 | 32 | 25 | 24 | 24 | 111 | 32 | 38 | 47 | 38 | 39 | 30 | 35 | 15 |
| Sierra Leone | 74 | 7 | 30 | 48 | 42 | 48 | 105 | 29 | 39 | 36 | 77 | 33 | 36 | 48 | 42 |
| Tanzania | 57 | 9 | 52 | 45 | 40 | 29 | 210 | 49 | 74 | 74 | 86 | 70 | 50 | 96 | 41 |
| The Gambia | 106 | 9 | 60 | 59 | 76 | 59 | 126 | 41 | 61 | 75 | 64 | 62 | 53 | 75 | 39 |
| Togo | 42 | 16 | 70 | 33 | 70 | 41 | 102 | 43 | 78 | 94 | 75 | 78 | 51 | 99 | 23 |
| Uganda | 15 | 7 | 24 | 36 | 39 | 45 | 109 | 24 | 25 | 38 | 32 | 27 | 30 | 34 | 28 |
|  |  |  |  |  |  |  |  |  |  |  |  |  |  |  |  |
| **AEZ** |  |  |  |  |  |  |  |  |  |  |  |  |  |  |  |
| Arid | 300 | 11 | 59 | 58 | 60 | 56 | 122 | 50 | 67 | 92 | 67 | 70 | 50 | 86 | 41 |
| Highlands | 169 | 12 | 74 | 59 | 37 | 61 | 172 | 46 | 113 | 139 | 82 | 113 | 65 | 86 | 29 |
| Humid | 106 | 13 | 70 | 74 | 40 | 68 | 124 | 50 | 85 | 105 | 86 | 83 | 40 | 118 | 43 |
| Sub-humid | 423 | 14 | 102 | 79 | 79 | 71 | 111 | 56 | 122 | 137 | 100 | 121 | 66 | 111 | 69 |
|  |  |  |  |  |  |  |  |  |  |  |  |  |  |  |  |
| **PS^a^** |  |  |  |  |  |  |  |  |  |  |  |  |  |  |  |
| IL | 326 | 9 | 67 | 76 | 57 | 79 | 133 | 46 | 78 | 88 | 63 | 78 | 46 | 93 | 35 |
| RL | 438 | 14 | 86 | 93 | 57 | 93 | 140 | 60 | 122 | 158 | 114 | 122 | 65 | 104 | 64 |
| RU | 234 | 14 | 61 | 81 | 60 | 83 | 103 | 50 | 81 | 113 | 111 | 80 | 56 | 111 | 50 |
|  |  |  |  |  |  |  |  |  |  |  |  |  |  |  |  |
| **Overall** | 998 | 13 | 85 | 87 | 65 | 90 | 130 | 55 | 107 | 129 | 94 | 107 | 60 | 108 | 58 |

**^a^**IL=irrigated lowland, RL=rainfed lowland, RU=rainfed upland. ^b^n denotes the number of farmer’s fields

**Table B3.** Coefficients of variation (%) of recorded mineral N, P, and K fertilizers application rates and grain yields of this study of “n” farmers’ fields for each country, across agro-ecological zones (AEZ) and production systems (PS), and overall.

| **Country/AEZ/PS** | **n^b^** | **N**  **(kg N ha^-1^)** | **P**  **(kg P ha^-1^)** | **K**  **(kg K ha^-1^)** | **Grain Yield**  **(ton ha^-1^)** |
| --- | --- | --- | --- | --- | --- |
| **Country** |  |  |  |  |  |
| Benin | 105 | 83 | 78 | 78 | 59 |
| Burkina Faso | 11 | 155 | 154 | 157 | 50 |
| Cameroon | 74 | 104 | 165 | 164 | 62 |
| Chad | 9 |  |  |  | 23 |
| Côte d’Ivoire | 69 | 271 | 324 | 315 | 53 |
| DRC | 24 |  |  |  | 37 |
| Ethiopia | 23 | 322 | 302 |  | 35 |
| Ghana | 97 | 77 | 110 | 124 | 53 |
| Guinea | 56 | 166 | 156 | 156 | 51 |
| Madagascar | 54 | 735 |  |  | 56 |
| Mali | 65 | 92 | 143 | 141 | 50 |
| Niger | 14 | 49 | 39 | 49 | 18 |
| Nigeria | 22 |  |  |  | 43 |
| Rwanda | 45 | 45 | 54 | 81 | 51 |
| Senegal | 36 | 43 | 264 |  | 44 |
| Sierra Leone | 74 | 741 | 741 | 741 | 44 |
| Tanzania | 57 | 185 | 755 |  | 46 |
| The Gambia | 106 | 149 | 134 | 134 | 55 |
| Togo | 42 | 132 | 128 | 128 | 28 |
| Uganda | 15 | 387 |  |  | 52 |
|  |  |  |  |  |  |
| **AEZ** |  |  |  |  |  |
| Arid | 36 | 99 | 141 | 123 | 55 |
| Highlands | 169 | 140 | 155 | 176 | 53 |
| Humid | 106 | 244 | 350 | 370 | 59 |
| Sub-humid | 423 | 182 | 179 | 184 | 63 |
|  |  |  |  |  |  |
| **PS^a^** |  |  |  |  |  |
| IL | 326 | 94 | 120 | 116 | 41 |
| RL | 438 | 173 | 234 | 251 | 66 |
| RU | 234 | 220 | 184 | 196 | 60 |
|  |  |  |  |  |  |
| **Overall** | 998 | 147 | 178 | 178 | 64 |

**^a^**IL=irrigated lowland, RL=rainfed lowland, RU=rainfed upland. ^b^n denotes the number of farmer’s fields

**Appendix C. Models Akaike Information Criterion (AIC), Nakagawa’s conditional [*R^2^* (cond.)] and marginal [*R^2^* (marg.)], and modeling efficiency (EF)**

**Table C1.** Akaike Information Criterion (AIC), Nakagawa’s conditional and marginal *R^2^*, modeling efficiency (EF) for mixed effects models explaining observed variations in concentrations of N, P, and K in rice grain

| **Model ID** | **Model composition** | **AIC** | ***R^2^* (cond.)** | ***R^2^* (marg.)** | **EF** |
| --- | --- | --- | --- | --- | --- |
| Mod N1 | AEZ | -523 | 0.30 | 0.11 | 0.219 |
| Mod N2 | AEZ+PS | -520 | 0.32 | 0.13 | 0.229 |
| Mod N3 | AEZ+PS+pH+N+TOC+Bray-P+Clay+  K-exc+Ca-exc+Mg-exc+Mn-exc+Cu+Bo+Al+CEC | -368 | 0.40 | 0.20 | 0.282 |
| Mod N4 | AEZ+PS+TOC | -509 | 0.33 | 0.13 | 0.229 |
| Mod N5 | AEZ+PS+Clay | -511 | 0.33 | 0.14 | 0.234 |
| Mod N6 | AEZ+PS+exc-Cations | -510 | 0.33 | 0.13 | 0.233 |
| Mod N7 | AEZ+PS+CEC | -510 | 0.33 | 0.14 | 0.233 |
| Mod N8 | AEZ+PS+N rate+P rate+K rate | -476 | 0.33 | 0.14 | 0.232 |
| Mod N9 | AEZ+PS+N | -515 | 0.33 | 0.14 | 0.230 |
| Mod N10 | AEZ+PS+N rate | -504 | 0.32 | 0.13 | 0.230 |
| Mod N11 | AEZ+PS+N+N rate | -499 | 0.33 | 0.14 | 0.232 |
|  |  |  |  |  |  |
| Mod P1 | AEZ | -3389 | 0.53 | 0.17 | 0.375 |
| Mod P2 | AEZ+PS | -3455 | 0.61 | 0.25 | 0.430 |
| Mod P3 | AEZ+PS+pH+N+TOC+Bray-P+Clay+  K-exc+Ca-exc+Mg-exc+Mn-exc+Cu+Bo+Al+CEC | -3208 | 0.61 | 0.24 | 0.459 |
| Mod P4 | AEZ+PS+TOC | -3441 | 0.61 | 0.25 | 0.430 |
| Mod P5 | AEZ+PS+Clay | -3439 | 0.61 | 0.25 | 0.431 |
| Mod P6 | AEZ+PS+exc-Cations | -3438 | 0.62 | 0.25 | 0.430 |
| Mod P7 | AEZ+PS+CEC | -3438 | 0.62 | 0.25 | 0.430 |
| Mod P8 | AEZ+PS+N rate+P rate+K rate | -3406 | 0.61 | 0.25 | 0.434 |
| Mod P9 | AEZ+PS+Bray-P | -3439 | 0.61 | 0.25 | 0.430 |
| Mod P10 | AEZ+PS+Al | -3448 | 0.61 | 0.25 | 0.432 |
| Mod P11 | AEZ+PS+Bray-P+Al+P rate | -3407 | 0.62 | 0.25 | 0.432 |
|  |  |  |  |  |  |
| Mod K1 | AEZ | -2804 | 0.67 | 0.14 | 0.488 |
| Mod K2 | AEZ+PS | -2832 | 0.71 | 0.20 | 0.513 |
| Mod K3 | AEZ+PS+pH+N+TOC+Bray-P+Clay+  K-exc+Ca-exc+Mg-exc+Mn-exc+Cu+Bo+Al+CEC | -2654 | 0.73 | 0.16 | 0.563 |
| Mod K4 | AEZ+PS+TOC | -2820 | 0.72 | 0.20 | 0.514 |
| Mod K5 | AEZ+PS+Clay | -2814 | 0.71 | 0.20 | 0.513 |
| Mod K6 | AEZ+PS+exc-Cations | -2841 | 0.75 | 0.20 | 0.527 |
| Mod K7 | AEZ+PS+CEC | -2837 | 0.74 | 0.20 | 0.525 |
| Mod K8 | AEZ+PS+N rate+P rate+K rate | -2779 | 0.71 | 0.20 | 0.514 |
| Mod K9 | AEZ+PS+K-exc | -2836 | -2836 | 0.72 | 0.20 |
| Mod K10 | AEZ+PS+K rate | -2815 | -2816 | 0.71 | 0.20 |
| Mod K11 | AEZ+PS+K-exc+K rate | -2820 | -2820 | 0.72 | 0.20 |

*R^2^* (marg.) is the marginal coefficient of determination showing the observed variations explained by the fixed factors in mixed-effects models

**Table C2.** Akaike Information Criterion (AIC), Nakagawa’s conditional and marginal *R^2^*, modeling efficiency (EF) for mixed effects models explaining observed variations in concentrations of Ca, Mg, S, and Fe in rice grain

| **Model ID** | **Model composition** | **AIC** | ***R^2^* (cond.)** | ***R^2^* (marg.)** | **EF** |
| --- | --- | --- | --- | --- | --- |
| Mod Ca1 | AEZ | -7127 | 0.27 | 0.07 | 0.178 |
| Mod Ca2 | AEZ+PS | -7112 | 0.29 | 0.11 | 0.187 |
| Mod Ca3 | AEZ+PS+pH+N+TOC+Bray-P+Clay+  K-exc+Ca-exc+Mg-exc+Mn-exc+Cu+Bo+Al+CEC | -6755 | 0.36 | 0.16 | 0.224 |
| Mod Ca4 | AEZ+PS+TOC | -7101 | 0.29 | 0.11 | 0.193 |
| Mod Ca5 | AEZ+PS+Clay | -7091 | 0.29 | 0.12 | 0.189 |
| Mod Ca6 | AEZ+PS+exc-Cations | -7093 | 0.28 | 0.11 | 0.188 |
| Mod Ca7 | AEZ+PS+CEC | -7093 | 0.28 | 0.11 | 0.188 |
| Mod Ca8 | AEZ+PS+N rate+P rate+K rate | -7048 | 0.29 | 0.11 | 0.191 |
| Mod Ca9 | AEZ+PS+Ca-exc | -7098 | 0.28 | 0.12 | 0.188 |
|  |  |  |  |  |  |
| Mod Mg1 | AEZ | -4970 | 0.47 | 0.09 | 0.412 |
| Mod Mg2 | AEZ+PS | -5051 | 0.67 | 0.25 | 0.477 |
| Mod Mg3 | AEZ+PS+pH+N+TOC+Bray-P+Clay+  K-exc+Ca-exc+Mg-exc+Mn-exc+Cu+Bo+Al+CEC | -4763 | 0.61 | 0.23 | 0.498 |
| Mod Mg4 | AEZ+PS+TOC | -5036 | 0.67 | 0.25 | 0.477 |
| Mod Mg5 | AEZ+PS+Clay | -5036 | 0.65 | 0.25 | 0.479 |
| Mod Mg6 | AEZ+PS+exc-Cations | -5034 | 0.66 | 0.26 | 0.477 |
| Mod Mg7 | AEZ+PS+CEC | -5034 | 0.66 | 0.26 | 0.477 |
| Mod Mg8 | AEZ+PS+N rate+P rate+K rate | -4991 | 0.67 | 0.25 | 0.478 |
| Mod Mg9 | AEZ+PS+Mg-exc | -5033 | 0.66 | 0.25 | 0.477 |
|  |  |  |  |  |  |
| Mod S1 | AEZ | -5963 | 0.50 | 0.16 | 0.380 |
| Mod S2 | AEZ+PS | -5938 | 0.52 | 0.18 | 0.382 |
| Mod S3 | AEZ+PS+pH+N+TOC+Bray-P+Clay+  K-exc+Ca-exc+Mg-exc+Mn-exc+Cu+Bo+Al+CEC | -5624 | 0.54 | 0.20 | 0.414 |
| Mod S4 | AEZ+PS+TOC | -5923 | 0.51 | 0.18 | 0.382 |
| Mod S5 | AEZ+PS+Clay | -5919 | 0.53 | 0.19 | 0.383 |
| Mod S6 | AEZ+PS+exc-Cations | -5924 | 0.52 | 0.18 | 0.385 |
| Mod S7 | AEZ+PS+CEC | -5923 | 0.53 | 0.19 | 0.385 |
| Mod S8 | AEZ+PS+N rate+P rate+K rate | -5875 | 0.52 | 0.18 | 0.382 |
|  |  |  |  |  |  |
| Mod Fe1 | AEZ | 13177 | 0.47 | 0.16 | 0.347 |
| Mod Fe2 | AEZ+PS | 13155 | 0.48 | 0.17 | 0.355 |
| Mod Fe3 | AEZ+PS+pH+N+TOC+Bray-P+Clay+  K-exc+Ca-exc+Mg-exc+Mn-exc+Cu+Bo+Al+CEC | 12909 | 0.57 | 0.29 | 0.414 |
| Mod Fe4 | AEZ+PS+TOC | 13151 | 0.47 | 0.16 | 0.355 |
| Mod Fe5 | AEZ+PS+Clay | 13156 | 0.48 | 0.17 | 0.355 |
| Mod Fe6 | AEZ+PS+exc-Cations | 13153 | 0.50 | 0.17 | 0.357 |
| Mod Fe7 | AEZ+PS+CEC | 13154 | 0.49 | 0.17 | 0.356 |
| Mod Fe8 | AEZ+PS+N rate+P rate+K rate | 13139 | 0.52 | 0.22 | 0.370 |

*R^2^* (marg.) is the marginal coefficient of determination showing the observed variations explained by the fixed factors in mixed-effects models

**Table C3.** Akaike Information Criterion (AIC), Nakagawa’s conditional and marginal *R^2^*, modeling efficiency (EF) for mixed effects models explaining observed variations in concentrations of B, Mn, and Cu in rice grain

| **Model ID** | **Model composition** | **AIC** | ***R^2^* (cond.)** | ***R^2^* (marg.)** | **EF** |
| --- | --- | --- | --- | --- | --- |
| Mod B1 | AEZ | 992 | 0.75 | 0.15 | 0.550 |
| Mod B2 | AEZ+PS | 983 | 0.74 | 0.17 | 0.559 |
| Mod B3 | AEZ+PS+pH+N+TOC+Bray-P+Clay+  K-exc+Ca-exc+Mg-exc+Mn-exc+Cu+B+Al+CEC | 955 | 0.80 | 0.19 | 0.646 |
| Mod B4 | AEZ+PS+TOC | 992 | 0.75 | 0.17 | 0.560 |
| Mod B5 | AEZ+PS+Clay | 996 | 0.75 | 0.18 | 0.560 |
| Mod B6 | AEZ+PS+exc-Cations | 942 | 0.79 | 0.19 | 0.584 |
| Mod B7 | AEZ+PS+CEC | 951 | 0.78 | 0.19 | 0.580 |
| Mod B8 | AEZ+PS+N rate+P rate+K rate | 996 | 0.75 | 0.18 | 0.574 |
| Mod B9 | AEZ+PS+B | 956 | 0.76 | 0.17 | 0.574 |
|  |  |  |  |  |  |
| Mod Mn1 | AEZ | 8305 | 0.46 | 0.05 | 0.400 |
| Mod Mn2 | AEZ+PS | 8296 | 0.49 | 0.05 | 0.406 |
| Mod Mn3 | AEZ+PS+pH+N+TOC+Bray-P+Clay+  K-exc+Ca-exc+Mg-exc+Mn-exc+Cu+B+Al+CEC | 8187 | 0.47 | 0.10 | 0.455 |
| Mod Mn4 | AEZ+PS+TOC | 8285 | 0.48 | 0.04 | 0.414 |
| Mod Mn5 | AEZ+PS+Clay | 8299 | 0.48 | 0.06 | 0.408 |
| Mod Mn6 | AEZ+PS+exc-Cations | 8299 | 0.50 | 0.05 | 0.408 |
| Mod Mn7 | AEZ+PS+CEC | 8299 | 0.50 | 0.05 | 0.408 |
| Mod Mn8 | AEZ+PS+N rate+P rate+K rate | 8307 | 0.50 | 0.05 | 0.414 |
| Mod Mn9 | AEZ+PS+Mn-exc | 8286 | 8292 | 0.49 | 0.05 |
|  |  |  |  |  |  |
| Mod Cu1 | AEZ | 7155 | 0.33 | 0.09 | 0.239 |
| Mod Cu2 | AEZ+PS | 7150 | 0.34 | 0.09 | 0.244 |
| Mod Cu3 | AEZ+PS+pH+N+TOC+Bray-P+Clay+  K-exc+Ca-exc+Mg-exc+Mn-exc+Cu+B+Al+CEC | 7019 | 0.55 | 0.26 | 0.352 |
| Mod Cu4 | AEZ+PS+TOC | 7138 | 0.37 | 0.11 | 0.256 |
| Mod Cu5 | AEZ+PS+Clay | 7157 | 0.33 | 0.09 | 0.244 |
| Mod Cu6 | AEZ+PS+exc-Cations | 7148 | 0.33 | 0.09 | 0.249 |
| Mod Cu7 | AEZ+PS+CEC | 7143 | 0.33 | 0.09 | 0.253 |
| Mod Cu8 | AEZ+PS+N rate+P rate+K rate | 7158 | 0.34 | 0.09 | 0.244 |
| Mod Cu9 | AEZ+PS+Cu | 7158 | 0.33 | 0.09 | 0.246 |

*R^2^* (marg.) is the marginal coefficient of determination showing the observed variations explained by the fixed factors in mixed-effects models
